# The Potential, Applications, and Challenges of Internet of Things (IoT) Technologies From the Perspectives of Clinicians

**DOI:** 10.7759/cureus.85847

**Published:** 2025-06-12

**Authors:** Abdullah Alanazi

**Affiliations:** 1 Department of Health Informatics, College of Public Health and Health Informatics, King Saud Bin Abdulaziz University for Health Sciences, Riyadh, SAU; 2 Health Informatics, King Abdullah International Medical Research Center, Riyadh, SAU; 3 Health Informatics, King Abdulaziz Medical City Riyadh, Riyadh, SAU

**Keywords:** access, devices, iot, monitoring, the internet of thing

## Abstract

Introduction

The introduction of the Internet of Things (IoT) in our lives ranges from simple tracking to smart homes. While the healthcare sector is still lagging, this study aims to explore the feasibility and challenges of incorporating IoT into the healthcare industry.

Method

This study employed a qualitative approach to gather clinicians' opinions from various healthcare professions. Data were collected, and consensus was reached through three rounds of discussions.

Results

Twenty-four participants were involved in this study, out of whom 45% were physicians. The participants agreed on the potential benefits of IoT, which include improved quality, accessibility, and efficiency of healthcare. They identified several potential applications of IoT, including patient monitoring, hospital and medication management, tracking devices, and telemedicine. However, some challenges related to regulations, infrastructure, the need for reforms in reimbursement, and concerns over patient privacy and data confidentiality were also noted.

Conclusion

Collaborative efforts should be initiated to enhance the adoption of IoT in healthcare. These efforts should not be limited to regulatory, legal, and technical aspects. Additionally, there should be a shift in perspective that extends healthcare services beyond the confines of healthcare organizations.

## Introduction

The Internet of Things (IoT) is a network of sensor-enabled physical devices that are connected to collect, exchange, and share data over the internet. This concept, first conceived by Kevin Ashton in 1988, has garnered significant attention from the healthcare industry [1]. The IoT has the potential to revolutionize access to healthcare services as well as the efficacy and efficiency of the healthcare system [2]. For instance, wearable devices can monitor personal health data and facilitate client communication [3]. Mobile health (mHealth) has also connected patients and healthy individuals to healthcare services and enabled healthcare outside hospitals [4].

In recent years, IoT technology has begun gaining significant attention in the healthcare industry, although it is still nascent. In addition to using wearable devices for general wellness, therapeutic purposes are introduced, and clinical-related data are shared with medical professionals through different IoT products [5].

However, not all the data collected by these devices carries the same therapeutic value; in addition, it is unclear whether all products of IoT should be registered and licensed by the Food and Drug Administration (FDA) [6]. Some low-risk IoT devices were exempt from the FDA licenses as they appear for wellness purposes. Hence, consumers need to carefully evaluate the accuracy of these devices and consult healthcare providers before making decisions based on the reading of these IoT devices [6,7].

It can be challenging for patients and healthcare practitioners to differentiate between clinical IoT devices and wellness gadgets, as the distinction between the two is becoming increasingly blurred with the growing popularity of personal wellness devices in healthcare [8]

The healthcare industry gains significant advantages by adopting the IoT as real-time data is acquired; healthcare providers can access the most current and accurate information about their patient's health, enabling them to respond quickly and improve overall outcomes [9]. Moreover, the IoT can automate data collection and analysis [10]. Remote care is also facilitated through the IoT, allowing patients to receive care in the comfort of their own homes while minimizing the need for in-person appointments [11]. IoT devices can bridge the digital divide by connecting patients living in underserved areas through IoT devices [12].

However, IoT technology in the healthcare industry may raise concerns regarding privacy and security, particularly when sensitive patient data is involved [13]. Compatibility and the ability to integrate IoT products with the current hospital information systems are among the main challenges for the broader adoption of IoT in healthcare. Further, complexity and user resistance are other challenges [14]. IoT devices can bridge the digital divide by connecting patients living in underserved areas through IoT devices. Considering the potential benefits and challenges surrounding the use of IoT in healthcare as discussed in the literature, this study aims to explore clinicians' perspectives on the potential, applications, and challenges associated with using IoT technologies in healthcare.

## Materials and methods

The study utilized qualitative data to explore clinicians' perspectives on adopting IoT in healthcare. Thus, the study was conducted using a three-round Delphi technique to address the study's objectives.

Delphi surveys can encompass panel sizes ranging from seven to 1,200 participants; however, De Loë (2016) recommends an optimal range of 10 to 50 participants [15]. An alternative perspective suggests that size is less critical, noting that no statistical power is required. Thus, a smaller panel size of 10 to 18 experts is often advocated. For this study, aimed at examining the benefits, applications, and challenges associated with the implementation of IoT in healthcare, a sample size of 30 individuals was selected to cover the main three themes of the study, allocating 10 clinicians per theme.

The study was conducted over three rounds to ensure thematic saturation and achieve consensus among the panel members. After each round, the investigators summarized the findings, coded them thematically, and shared them with the participants before moving to the next round, enabling them to adjust their answers based on the group's feedback. A third reviewer was invited in case of disagreement between the two investigators to ensure consistency in recording the responses.

The participants were selected purposefully, as exposure and interest in technology are the criteria that must be included in the study. Further, at least one year of clinical experience was also required. The participants were invited, and all communications were conducted in English. No compensation was provided to the participants for their participation. The tool used for data collection was a survey with open-ended questions and prompts to investigate IoT-related topics, including initial impressions and potential applications. The survey is attached in the Appendix. An evaluation was conducted in the third round, during which panelists reviewed the aggregated responses and re-evaluated their original answers.

The process was executed professionally, ensuring thorough documentation of all participant perspectives. Furthermore, the study adhered strictly to ethical standards, having obtained approval from the Institutional Review Board at King Abdullah International Medical Research Center (KAIMRC) (approval number IRB/1895/23) before data collection.

## Results

Data collection was done in three rounds to explore clinicians’ perceptions of IoT applications and the associated challenges. To address the issue of non-response, particularly in the later rounds, we purposively targeted well-recognized experts, providing each with a clear explanation of the value of their input. Regular follow-up communication was maintained, and we consistently expressed our appreciation for their valuable time and contributions throughout the process.

The principal investigator invited 33 participants, of whom 30 agreed to take part in the study. Ultimately, 24 participants completed all three rounds of discussions. The sociodemographic characteristics of the participants are summarized in Table 1. Thirteen participants (54%) were men, and 22 (91%) were over the age of 30 years. Participants represented a range of professional backgrounds: 11 (42%) were physicians, four (23%) were nurses, three (15%) were pharmacists, and six (19%) were allied healthcare providers. Only one participant (4%) had five years or less of clinical experience, while the majority reported having six or more years of experience. In terms of familiarity with electronic health records (EHRs), 23 participants (96%) rated their experience as intermediate or expert. Similarly, 23 participants (96%) reported feeling comfortable using EHRs, indicating moderate to high levels of confidence.

**Table 1:** The Sociodemographic Characteristics of the Participants EHR: electronic health records.

Variable	Responses	Frequency n (%)
Gender	Male	13 (54.2)
	Female	11 (45.8)
Age	20-29	2 (8.3)
	30-39	11 (45.8)
	40- older	11 (45.8)
Specialties	Physicians	11 (45.8)
	Nurses	4 (16.7)
	Pharmacists	3 (12.5)
	Laboratory Specialists	3 (12.5)
	Radiology Specialists	3 (12.5)
Clinical experience (yr)	Five or less	1 (4.2)
	6-10	7 (29.2)
	11-15	10(41.7)
	Over 15	6 (25)
EHR Experience (years)	Novice <3	1 (4.2)
	Intermediate 3-5	5 (20.8)
	Expert >5	18 (75)
Comfort levels with EHR	Low	1 (4.2)
	Moderate	10 (33.3)
	High	13 (54.2)

Intercoder reliability was assessed by having two coders independently code the participants’ responses. If the two coders could not reach a consensus, a third coder was consulted. Establishing intercoder reliability is essential to ensure a systematic and transparent coding process. Further, to reach a consensus on the thematics ranking, the level of agreement was calculated based on the approach by Anthony (1999) [16], which defines agreement levels using K-values (Table 2).

**Table 2:** The Agreement-level Scale

K value	Agreement level
0.00-0.20	Poor
0.21-0.40	Fair
0.41-0.60	Moderate
0.061-0.80	Substantial
0.81-1	Almost perfect

Additionally, the level of importance was assessed throughout the rounds using a five-point Likert scale, ranging from "Strongly disagree" to "Strongly agree".

The participants consistently identified and prioritized key clinical advantages linked to the adoption of IoT in their daily practice. These advantages, refined through iterative feedback and collective appraisal, are comprehensively summarized in Table 3. 

**Table 3:** The Perceived Benefits of Internet of Things (IoT)

Themes of Benefits of IoT	Frequency, n (%)
Improve the quality of care	22 (91.7)
Improve access to care and monitoring	21 (87.5)
Enhance resources utilization	17 (70.8)
Cost saving	11 (45.8)

For example, the agreement and importance values for the first theme under the perceived benefits of IoT are presented in Table 4.

**Table 4:** The Agreement and Importance Values of the First Answer in the Perceived Benefit of Internet of Things (IoT)

K value	Agreement level	Round 1	Round 2	Round 3
Agreement	Strongly agree	42%	46%	54%
	Agree	46%	54%	46%
	Neutral	8%	0%	0%
	Disagree	4%	0%	0%
	Strongly disagree	0%	0%	0%
Importance	Median	3	2	2
	Range	2-6	1-4	1-3

These values reflect both the level of agreement among participants and how important they perceived this theme across the three rounds.

On the second aspect of the study, the expert panel reached a consensus concerning the perceived potential of IoT technologies within clinical practice. Participating clinicians systematically identified, evaluated, and prioritized key clinical applications of IoT across various healthcare environments throughout a structured three-round Delphi process. The consolidated outcomes of this iterative consensus methodology are presented in Table 5.

**Table 5:** The Perceived Applications of IoT

Themes of applications of IoT	Frequency, n (%)
Remote Patient Monitoring	22 (91.7)
Telemedicine	20 (83.3)
Wearable Health Devices	18 (75)
Medication Management	17 (70.8)
Hospital Management	13 (54.2)
Asset and Inventory Management	11 (45.8)

The expert panel reached a consensus on the key challenges associated with adopting IoT technologies in healthcare. One participant raised concerns related to cost, connectivity, and device accuracy, stating, “I can see issues related to cost, connection, and trust in the accuracy of IoT devices.” Another participant emphasized the importance of data security, noting that “Patient data should be carefully preserved and properly maintained.” A third participant highlighted concerns around regulation and reimbursement, remarking, “I wonder who would compensate me and even how I would be held accountable for following up with patients beyond my clinic.” The finalized results, obtained through a structured and iterative consensus-building process, are detailed in Table 6.

**Table 6:** The Perceived Challenges of Internet of Things (IoT) Technology

Themes of Challenges of IoT Technology	Frequency, n (%)
Privacy and Security	23 (95.8)
Interoperability Issues	19 (79.2)
High Implementation Costs	17 (70.8)
Complexity of Deployment	17 (70.8)
Regulatory and Compliance Challenges	11 (45.8)
Patient-Generated Data Management and Analysis	10 (41.7)
Ethical and Legal Issues	9 (37.5)

Table 7 provides examples of representative quotes from the participants, aligned with the themes identified in the study.

**Table 7:** Representative Quotes of the Participants

Themes	Representative Quotes
Perceived benefit of IoT	I believe IoT could really help me track and monitor my patients while they’re at home. It would allow me to extend care beyond the hospital setting and stay connected with patients remotely. IoT could also make it easier for me to keep track of medical stock and help reduce the chances of drugs expiring before they’re used.
Perceived applications of IoT	I think IoT could really help me provide care to patients remotely. It might reduce the need for hospital admissions. I believe IoT can also make managing hospital assets much easier.
Perceived challenges of IoT	I can see issues related to cost, connection, and trust in the accuracy of IoT devices. Patient data should be carefully preserved and properly maintained. I wonder who would compensate me and even how I would be held accountable for following up with patients beyond my clinic.

## Discussion

The idea of connecting patients with healthcare providers beyond the physical boundaries of healthcare premises has constantly attracted healthcare leaders. Many scholars and researchers have studied possible technologies that enable this connection. The clinicians enrolled in this study have discussed the potential benefits, applications, and challenges of introducing IoT devices in healthcare. 

An expert panel has agreed on several key advantages of adopting IoT in healthcare; these advantages span from the role of IoT in enabling remote monitoring of patients, the role of IoT in conducting care sessions across distances through telemedicine, and the expected role of IoT in wearable devices or textile for monitoring health and well-being of individuals. Additionally, the expert has emphasized the anticipated role of IoT in medication management, hospital management, and asset and inventory management.

The role of IoT technology can go beyond the monitoring of vital signs; it can be utilized even for early detection of disease and complications, enabling the healthcare team to make prompt and personalized interventions. This finding aligns with the consensus in most literature [17,18]. Further, consistent with previous studies, IoT technologies can improve resource utilization and streamline routine processes in healthcare; for instance, they can be used to track the devices in the hospital, for stock management, and for routine maintenance [3,19,20]. Furthermore, IoT can be used to provide healthcare services, particularly in remote and underserved communities, and these advantages of IoT were described in other studies [18,21]. This finding is similar to a study conducted by Taherdoost who noted that wearable and implantable IoT devices enable the continuous tracking of vital signs and other health indicators in patients with chronic diseases such as diabetes, hypertension, and cardiovascular conditions [22]. Another prominent application, as stated by our panel, is in telemedicine, where IoT devices facilitate remote consultations and diagnostic procedures by transmitting clinical data in real time. Similarly, Lee et al. conducted a study and observed that using devices such as smart stethoscopes, otoscopes, and ECG monitors contributes to accurate, remote clinical assessments and the development of appropriate treatment plans [23]. Beyond equipment management, IoT devices regulate environmental parameters such as temperature, humidity, and air quality, ensuring that healthcare settings remain safe and conducive to patient recovery. Further, the Man et al. study focuses on IoT-based asset management [24].

Despite these advancements, our panel identified several challenges that hinder the widespread adoption of IoT in healthcare. Privacy and security concerns remain paramount, as sensitive personal health data are generated and shared over IoT devices [12]. Establishing security measures and adhering to privacy regulations are essential prerequisites for safeguarding patient data and building public trust in these technologies [6,12]. Interoperability is hindered by the lack of agreed-upon standards and protocols, which pose a significant challenge due to the wide variety of devices, vendors, purposes, and data. Another challenge is the initial costs of acquiring IoT devices and building the supporting infrastructure [25].

Additionally, device maintenance, upgrades, and the required cost of security measures further overwhelm the already high cost of healthcare systems. The complexity of some IoT devices may demand specialized technical expertise that healthcare technicians may not possess. Implementing and integrating the IoT devices necessitates training targeting clinicians, consumers, and technical staff to ensure proper adoption and effective utilization of IoT [26]. Not providing training to the clinicians and staff and showing them the value of investing time in using IoT and analyzing the generated data is another solution to overcome the culture barriers and staff resistance, and this was discussed in an early study by Kumar et al. who found that IoT can increase workload and may disturb the routine of the staff [27]. The vast and versatile nature of data generated by the IoT device requires robust data management systems and proper analytical capabilities, as these challenges can compromise the value of using IoT in the clinical decision-making process. This challenge was discussed by Singh et al. [28]

Regulatory and compliance-related challenges also complicate IoT adoption. Devices utilized in healthcare must undergo rigorous testing and obtain approvals from regulatory agencies such as the Food and Drug Administration (FDA) [29]. Some IoT devices utilised for well-being do not qualify for therapeutic uses. However, compliance with regulations and the establishment of new specific regulations are necessary to ensure patient safety and build trust among providers and patients [29]. Some ethical and legal issues remain unresolved, particularly regarding data ownership, breaches, and liability in cases of device malfunctions [30].

Study recommendations

IoT technology has the potential to transform the healthcare system by improving the quality of care, accessibility, and efficiency of healthcare services. However, achieving this transformation requires involvement from clinicians, healthcare executives, policymakers, vendors, and patients. Collaboration between healthcare executives and policymakers is essential to establish a regulatory framework that appropriately distinguishes between therapeutics and wellness. Additionally, it is crucial to develop the necessary infrastructure, implement robust security measures, and provide proper patient education and resources. Clinicians should take the initiative to analyze the data generated by IoT technology, incorporate insights cautiously, and make informed decisions to enhance patient health. Insurance companies and third-party payers must support the integration of IoT technology and recognize the value it adds by providing reimbursement for the efforts involved.

Study limitations

This study is qualitative and exploratory, which means it has certain limitations. It is inherently non-confirmatory and reflects the opinions of the participants involved. As a result, any generalizations should be made with caution. Additionally, there may be instances of groupthink and presentation bias. It is also important to note that IoT, like other emerging technologies, may become obsolete. Nevertheless, the IoT is an essential tool that connects patients and extends care beyond the confines of healthcare organizations.

## Conclusions

This study explores various aspects of IoT technology in healthcare. The perceived benefits include improved quality of care, increased access to healthcare and monitoring, enhanced resource utilization, and cost savings. The study identifies several potential applications of IoT, such as remote patient monitoring, telemedicine, wearable health devices, medication management, hospital management, and asset and inventory management.

However, incorporating IoT into healthcare presents several challenges. These include privacy and security concerns, interoperability issues, high implementation costs, the complexity of deployment, regulatory and compliance challenges, management and analysis of patient-generated data, and ethical and legal issues. A collaborative effort is essential for IoT to have a meaningful impact on healthcare.

## Appendices

**Table 8:** The Study Survey Details Developed by A. Alanazi

Details of the Survey	
Number of Participants	
Describe group composition (respondents' Information):	
Gender:	
Age:	
Background:	
Experience:	
Comfort level of Information Technology:	
List of Respondents; Respondent's Serial Number	
Before the discussion	
Inform respondents about the purpose and goal of the focus group discussion.	✔ ✖
Stress confidentiality to ensure that respondents' details, ideas and insights will be kept for the purpose of the focus group discussion.	✔ ✖
Have respondents introduce themselves to the group.	✔ ✖
Discussion	
How familiar are you with Internet of Things (IoT) technologies, challenges, and other issues from clinicians’ perspectives?	
When you think about Internet of Things (IoT) technologies, what would you think of possible applications? Impacts? Enhanced services?	
When you think about Internet of Things (IoT) technologies, what would you think of possible challenges? barriers? Possible threats?	
